# Decoding the antihypertensive mechanism of cannabidiol through integrative bioinformatics and machine learning

**DOI:** 10.1186/s42238-026-00437-5

**Published:** 2026-04-28

**Authors:** Xin Jiang, Ziyue Zhou, Yuqin Liang, Yu Wang, Shumin Liu, Fang Lu

**Affiliations:** 1 Institute of Traditional Chinese Medicine, Heilongjiang University of Chinese Medicine, Harbin, 150040 China; 2 First Clinical Medical College of Heilongjiang University of Chinese Medicine, Harbin, 150040 China

**Keywords:** Cannabidiol, Hypertension, Bioinformatics, Machine learning, Molecular dynamics simulation

## Abstract

**Background:**

Hypertension (HTN) results from intricate molecular mechanisms, making clinical remission difficult to achieve. This study explores the molecular pathways through which cannabidiol (CBD) may influence HTN.

**Methods:**

Several RNA sequencing datasets related to HTN were retrieved from the GEO database and divided into training and validation sets. Candidate genes potentially associated with HTN were screened through differential expression analysis and weighted gene co-expression network analysis. The interactions and binding potential between CBD and key target proteins were then systematically investigated using bioinformatics, machine learning, immune cell infiltration analysis, and molecular dynamics simulation.

**Result:**

Seventy genes were identified as potential targets for CBD intervention in HTN. Machine learning analysis refined this list to five core genes: pyruvate kinase PKM (PKM), thyroid hormone receptor beta (THRB), aldo–keto reductase family 1 member B1 (AKR1B1), TGF-beta receptor type-1 (TGFBR1), and proto-oncogene tyrosine-protein kinase Src (SRC). Among these, PKM, THRB, AKR1B1, and SRC were significantly upregulated in HTN, while TGFBR1 was downregulated (*P* < 0.05). These genes formed a regulatory network, showing direct or indirect interactions, and were associated with infiltration levels of neutrophils and resting mast cells. Molecular dynamics simulation revealed that CBD exhibits strong binding specificity to these target proteins.

**Conclusion:**

This integrated analysis prioritized PKM, THRB, AKR1B1, TGFBR1, and SRC as candidate genes potentially associated with HTN progression. Molecular dynamics simulation suggested a favorable binding potential between CBD and these targets. These findings may provide supportive evidence for future studies exploring the potential mechanisms by which CBD may act in HTN.

## Background

Hypertension, characterized by persistently elevated arterial blood pressure, is a chronic cardiovascular disease and a major global risk factor for myocardial infarction, stroke, and heart failure. Its pathogenesis is complex, involving overactivation of the sympathetic nervous system, endothelial dysfunction, chronic inflammation, and an imbalance of oxidative stress (Hu et al. 2025). Therefore, multi-target and multi-pathway intervention strategies are crucial for hypertension treatment. Cannabidiol, a non-psychoactive phytocannabinoid, exerts cardiovascular protective effects by modulating vascular tone, inhibiting inflammation, and reducing oxidative stress through multiple targets (Sultan et al. 2017).

Although preclinical studies consistently demonstrate the antioxidant, anti-inflammatory, and endothelial protective properties of cannabidiol (CBD), its regulation of blood pressure in humans exhibits significant "context-dependent" characteristics. In normotensive individuals, CBD has a minimal impact on resting blood pressure but significantly reduces stress-induced increases in blood pressure and improves arterial stiffness (Sultan et al. 2020). A meta-analysis confirmed this "stress-buffering" effect, indicating that CBD mainly inhibits sympathetically driven blood pressure elevation without significantly altering resting hemodynamics (Sultan et al. 2017). In contrast, in untreated hypertensive patients, acute CBD administration reduced 24-h systolic blood pressure by approximately 5 mmHg and mean arterial pressure by 3 mmHg, with more pronounced effects at night and improved arterial stiffness (Dragun et al. 2023). The landmark trial further confirmed that bioavailability-optimized CBD treatment sustainably lowered blood pressure and reduced angiotensin II levels (Kumric et al. 2023). Moreover, the antihypertensive effects of CBD plateau at moderate doses, with higher doses offering no additional benefits (Dujic et al. 2024). This reveals a critical differential response: CBD effectively mitigates sympathetic-mediated increases in blood pressure, including during acute stress and chronic hypertension, while having minimal impact on normal resting hemodynamics. This significant buffering effect on neurogenic hypertension, in contrast to its limited influence on baseline vascular tone regulation, highlights the precise context-dependency of CBD's vascular effects. The underlying molecular and physiological mechanisms remain unclear. Elucidating these state-dependent signaling pathways is essential for advancing therapeutic strategy development, guiding clinical medication use, and patient stratification.

This study aimed to systematically unravel the mechanisms underlying CBD's role in hypertension intervention using a multi-level systems biology approach. We integrated network pharmacology with multi-omics data to construct and analyze the action network of CBD. Key targets were identified through topological analysis and functional enrichment, while machine learning prioritized the core hub molecules. Additionally, the CIBERSORT method was used to quantitatively assess the infiltration levels of hypertension-related immune cells, identifying key immune cell types closely associated with core genes. Finally, molecular docking simulations verified the binding modes and interaction thermodynamic properties between potential targets and CBD. This research sought to elucidate the multi-scale regulatory network of CBD in hypertension intervention, providing a systematic basis for identifying its potential therapeutic targets.

## Methods

### Acquisition of disease-related targets

**Fig. 1:**
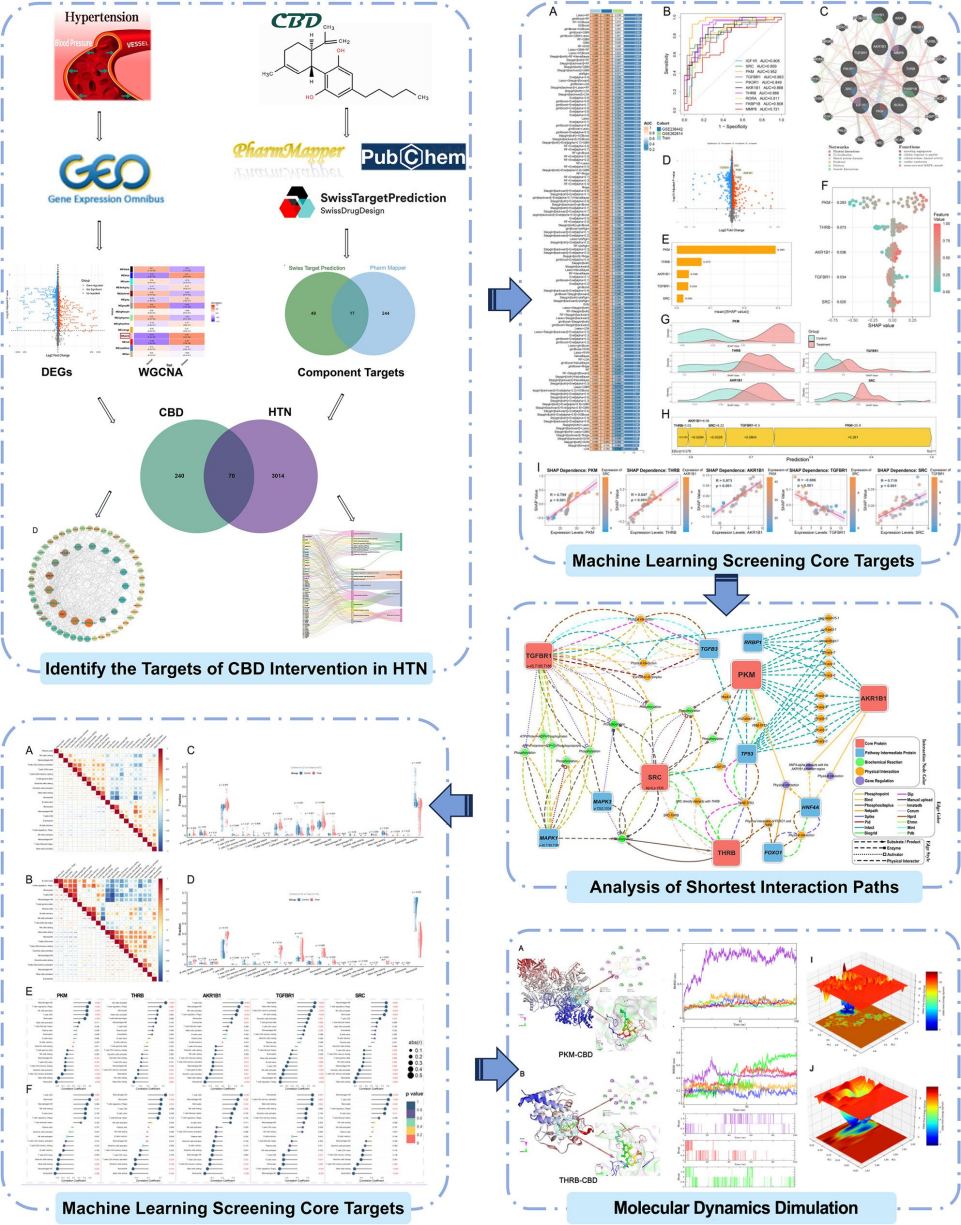
Flow-chart of dataset analysis in this paper

Four GEO transcriptomic datasets related to hypertension, GSE234085, GSE236442, GSE308365, and GSE262814, were included in this study. GSE234085, GSE236442, and GSE308365 were used for model development, whereas GSE262814 was used as the independent external validation cohort. In addition, because GSE236442 contained clinically distinct hypertension phenotypes, its refractory hypertension subgroup was analyzed separately as a phenotype-stratified supplementary validation set. The training datasets were merged after preprocessing, and batch effects were corrected using the SVA and ComBat algorithms. Subsequent analyses, including differential expression analysis, WGCNA, machine learning model construction, immune infiltration analysis, and molecular docking, were performed based on the model-development cohort, followed by validation in the independent external cohort and the phenotype-stratified supplementary cohort. The complete analysis workflow is shown in Fig. 1.

### Acquisition of active components and targets of CBD

The 2D structure of CBD was retrieved from the PubChem database, and potential targets were predicted using SwissTargetPrediction (http://www.swisstargetprediction.ch) and PharmMapper (http://lilab-ecust.cn/pharmmapper). All predicted targets were restricted to the Homo sapiens proteome, and annotation curation was performed using the UniProt database.

### Differential gene expression analysis

Transcriptome data were analyzed using the limma software package. Differentially expressed genes(DEGs) were identified by applying thresholds of an FDR adjusted *P* value < 0.05 and |log2 FC|> 0.585, with results visualized using ggplot2.

### Weighted gene co-expression network analysis (WGCNA)

Weighted gene co-expression network analysis (WGCNA) was performed on the batch-corrected expression matrix after normalization and variance filtering. Genes with low variance were removed, and the remaining expression matrix was used for sample clustering, soft-threshold selection, network construction, and module detection. A scale-free topology criterion was applied during soft-threshold selection, and gene modules were identified using dynamic tree cutting with a minimum module size of 30, followed by module merging at a cut height of 0.25. Module–trait relationships were evaluated by correlating module eigengenes with the hypertension phenotype using Pearson's correlation. The module showing the strongest association with hypertension was defined as the key disease-associated module and selected for downstream analysis. Differentially expressed genes (DEGs) were identified using the limma package with thresholds of FDR-adjusted *P* < 0.05 and |log2FC|> 0.585. To construct a comprehensive hypertension-related candidate pool, DEGs were merged with all genes from the key hypertension-associated WGCNA module after removal of duplicated gene symbols. The resulting nonredundant disease-related gene set was subsequently intersected with predicted CBD targets to identify candidate genes potentially involved in CBD-mediated regulation of hypertension.

### Identification of CBD-related disease targets and functional enrichment analysis

To identify core action targets of CBD in hypertension, DEGs were integrated with WGCNA module genes and subsequently intersected with predicted CBD targets. The overlapping genes were visualized using a Venn diagram. Gene Ontology (GO) and Kyoto Encyclopedia of Genes and Genomes (KEGG) pathway analyses were then performed using the Database for Annotation, Visualization and Integrated Discovery (DAVID) to elucidate potential mechanisms underlying CBD in hypertension pathogenesis.

### Machine learning for screening core genes

To prioritize CBD-related diagnostic biomarkers for hypertension, a multi-algorithm machine-learning framework was established based on the discovery cohort. Following integration of gene-expression matrices across datasets and batch correction using the ComBat algorithm, candidate genes derived from the upstream biological screening workflow were used to construct the machine-learning input matrix. The final dataset used for machine learning analysis comprised 64 samples from the model development cohort and 31 from the validation cohort. Twelve machine-learning algorithms were evaluated, including Enet, GBM, glmBoost, Lasso, LDA, Ridge, Naive Bayes, plsRglm, RF, Stepglm, SVM, and XGBoost. Predefined combinations of these algorithms were used to generate 127 candidate models for comparative analysis. The upstream biological screening pipeline first restricted candidate features, and algorithm-specific variable selection was then performed on the training matrix before final model construction. For model tuning, internal resampling strategies were implemented within several algorithms, including tenfold cross-validation in glmnet and GBM, k-fold resampling in glmBoost, and fivefold resampling in XGBoost. The training matrix was standardized before model fitting, and the validation matrix was standardized separately according to cohort designation. Model performance was assessed rigorously using the area under the receiver operating characteristic curve (AUC), accuracy, and F1 score. Subsequently, optimal single-model predictions were integrated via a stacking ensemble learning strategy. Models with high confidence (AUC > 0.9) were identified, and candidate core genes were determined by ranking feature genes based on their occurrence frequencies. The pheatmap package was used to visualize gene expression patterns, while PPI network analysis was conducted on the Gene MANIA platform.

### Model interpretability

Because machine learning models are inherently "black-box," this study employed the SHAP (Shapley Additive exPlanations) algorithm to quantify each feature's contribution to prediction outcomes. The method assigned a SHAP value to each feature, enabling an interpretable assessment of its impact on the model's predictions.

### Analysis of shortest interaction paths

ConsensusPathDB (http://cpdb.molgen.mpg.de/) is an online database that integrates information on gene regulatory networks, signaling pathways, metabolic processes, protein–protein interactions, and genetic interactions. Its ConPath tool was utilized to identify the shortest interaction paths connecting genes, and the resulting interaction networks were visualized using Cytoscape.

### Immune infiltration and functional analysis

Dynamic characteristics of immune cells in hypertension were assessed using the CIBERSORT deconvolution algorithm, a method based on linear support vector regression that systematically deconstructs cellular heterogeneity from complex gene expression matrices.

### Molecular dynamics simulation analysis

Molecular docking simulations were performed to validate interactions between CBD and the identified core genes. Molecular configurations were sourced from the PubChem and RCSB PDB databases. Protein structures were preprocessed by removing water molecules and adding hydrogen atoms. Ligand molecules were geometrically optimized using Chem3D 23.1.1 (MMFF94 force field, energy minimization convergence threshold of 0.01 kcal/(mol·Å)). Docking was performed with CB-Dock2, and results were analyzed and visualized in Discovery Studio 4.5. To further assess the dynamic behavior of the docked complexes, 100-ns molecular dynamics (MD) simulations were performed for the five CBD–protein complexes. Each system was subjected to standard preparation procedures, including protonation, hydrogen addition, solvation, ion neutralization, energy minimization, and equilibration under constant-volume and constant-pressure conditions, followed by production simulation under physiological temperature and pressure. Structural stability and conformational behavior were evaluated using root mean square deviation (RMSD), hydrogen-bond analysis, and principal component analysis (PCA)-based free energy landscape (FEL). Additional dynamic parameters, including root mean square fluctuation (RMSF) and radius of gyration (Rg), were calculated and are provided in the Supplementary Materials.

## Results

### Identification of hypertension-related target genes

**Fig. 2:**
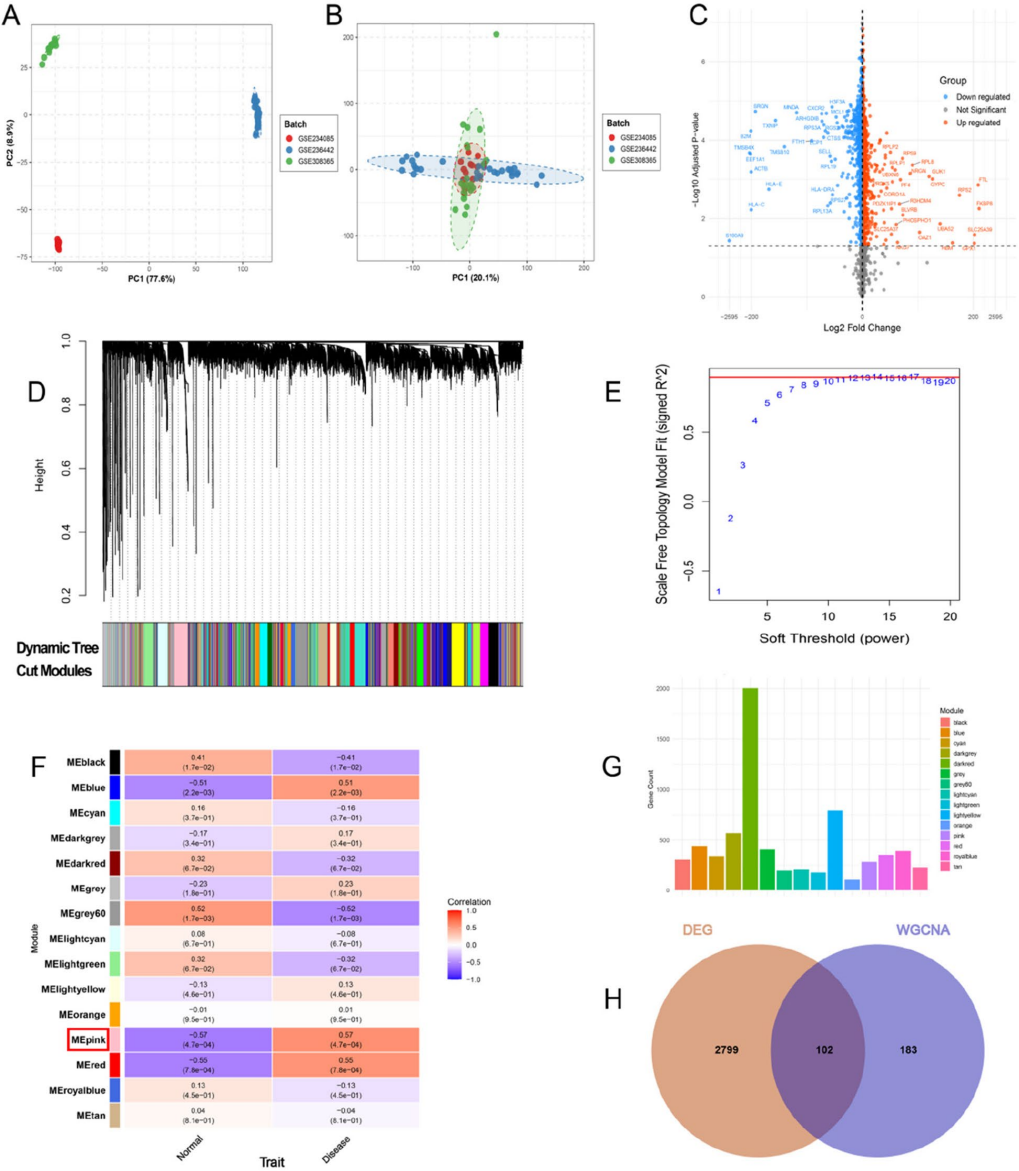
Identification of HTN-related target genes. **A**, **B** PCA plots for the GSE234085, GSE236442, and GSE308365 datasets before and after batch correction. **C** Volcano plot of differentially expressed genes (DEGs); red indicates upregulated DEGs, blue indicates downregulated DEGs, and gray indicates non-differentially expressed genes. **D** WGCNA gene dendrogram showing hierarchical clustering by co-expression, with module colors displayed at the bottom. **E** Scale independence analysis; the red horizontal line denotes the threshold. **F** Module–trait relationship heatmap depicting correlations between specific modules and HTN; numbers in the boxes are the correlation coefficients and *P* values. **G** The gene count statistics of the module. H Venn diagram showing the overlap between DEGs (orange) and WGCNA module genes (purple)

The GSE234085, GSE236442, and GSE308365 datasets were merged, and comprehensive normalization was applied to the combined gene expression matrix to mitigate batch effects. Principal component analysis (PCA) showed a marked improvement in data distribution after normalization, and the normalized datasets exhibited clearer clustering (Fig. 2A & B). Differential expression analysis identified 2901 genes with significant alterations in hypertension; these changes were displayed in a volcano plot (Fig. 2C). For WGCNA, the optimal soft-thresholding power (β) was first determined to ensure a scale-free network topology. Systematic evaluation of β values from 1 to 20 indicated that β = 9 was the minimum value meeting the scale-free topology criterion (R^2^ ≥ 0.8) (Fig. 2E). Using this parameter, a TOM was constructed, and hierarchical clustering was performed to identify co-expression modules, resulting in 15 distinct gene modules color-labeled for visualization (Fig. 2D). Module–trait relationship analysis revealed significant associations between specific modules and hypertension (R = 0.57, *P* = 4.7e-04) (Fig. 2F), with the pink module showing the strongest correlation in the module eigengene analysis. Gene count statistics analysis revealed the number of genes contained in each obtained co-expression module (Fig. 2G). By integrating DEGs with module eigengene results and removing duplicates, 3084 hypertension-related genes were ultimately identified (Fig. 2H).

### Identify the potential target proteins of CBD intervention in HTN

**Fig. 3:**
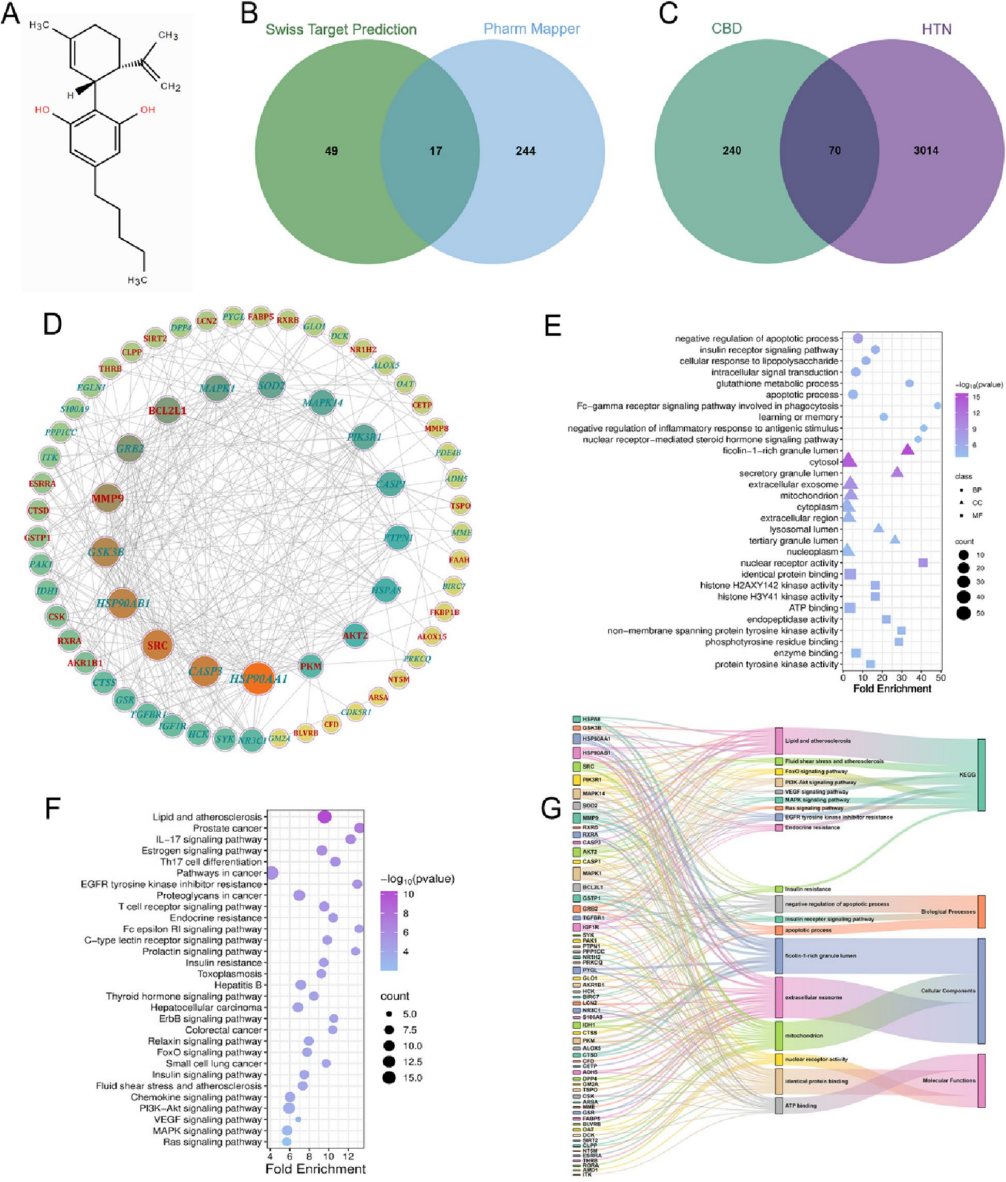
Identification of potential target proteins. **A** Chemical structure of CBD. **B** Venn diagram showing predicted target proteins from PharmMapper (green) and SwissTargetPrediction (blue). **C** Potential target proteins for CBD intervention in HTN. **D** PPI network of 70 potential targets. **E** GO enrichment analysis. **F** KEGG enrichment analysis. **G** Sankey diagram of enrichment results

The molecular structure of CBD was retrieved from the PubChem database (Fig. 3A). Following data integration from PharmMapper and SwissTargetPrediction and removal of redundant entries, a total of 310 CBD target proteins were identified (Fig. 3B). Intersection analysis with genes related to HTN yielded 70 potential targets implicated in CBD regulation of HTN. These targets were subjected to PPI analysis (Fig. 3C & D).

Comprehensive functional characterization was performed through GO and KEGG enrichment analyses (Fig. 3E & F). GO enrichment highlighted biological processes such as regulation of the apoptotic process, molecular functions including ficolin-1-rich granule lumen, and cellular components such as nuclear receptor activity. KEGG enrichment identified pathways of pathogenesis, including Lipid and atherosclerosis and Fluid shear stress and atherosclerosis; it also implicated key signaling pathways (FOXO, PI3K-Akt, VEGF, MAPK) and mechanisms of therapeutic resistance (EGFR tyrosine kinase inhibitor resistance, Endocrine resistance, Insulin resistance). Together, these results indicate that immune and endocrine factors, along with regulation of apoptosis and energy metabolism, play important roles in the mechanism of CBD intervention in HTN (Fig. 3G).

### Identification of core genes for CBD intervention in HTN

**Fig. 4:**
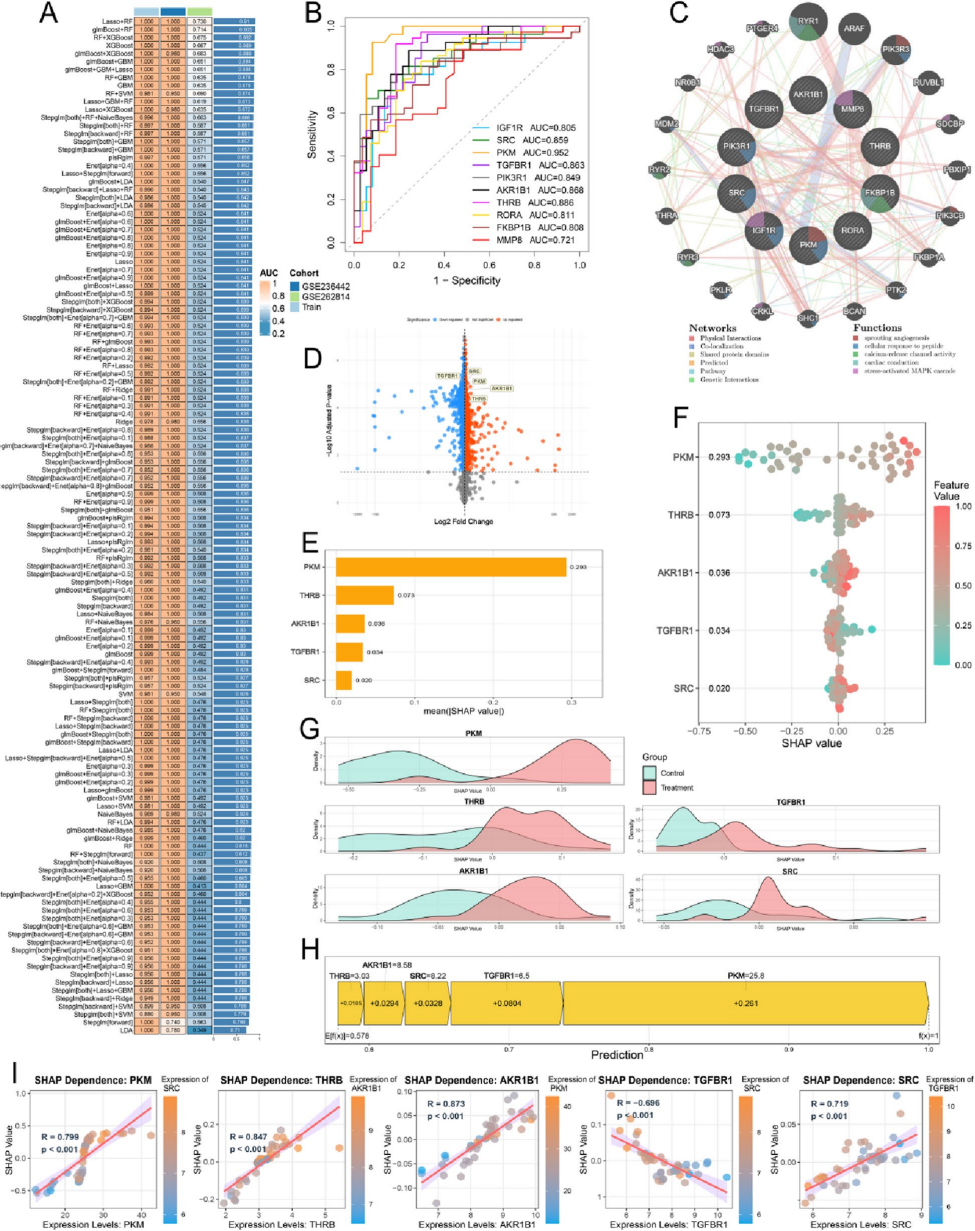
Identification of core genes through which CBD regulates HTN. **A** Comparison of model performance. A heatmap shows AUC values for each model across cohorts; higher values indicate better performance, and colors indicate the cohort source. **B** ROC curve for the candidate genes predicted by the optimal model. The x-axis shows the false positive rate, and the y-axis shows sensitivity; the AUC value indicates predictive performance. **C** PPI network of the characteristic genes, the ten genes inside the inner circle are the characteristic genes. **D** Volcano plot of DEGs; red indicates upregulated DEGs, blue indicates downregulated DEGs, and gray indicates non-differentially expressed genes. Core genes are marked. **E** Feature importance ranking. A bar chart lists core genes by feature importance; longer bars indicate greater contribution to the model. **F** A swarm plot shows gene expression distributions under different conditions. The plot width indicates data density, and color denotes expression level. **G** A distribution density plot displays the SHAP values of the core genes. Green denotes the Control group and red denotes the Treatment group; the plot indicates each gene’s contribution direction to the predictions. Negative SHAP values indicate inhibitory effects, and positive SHAP values indicate promoting effects. **H** Visualization of the contribution of core candidate genes to hypertension risk prediction. Force-directed analysis quantifies the contribution weight of each core candidate gene. **I** SHAP value distribution: A scatter plot displays the SHAP values of the key genes, revealing their impact on predictions. A color gradient represents the response intensity of core gene expression

Comprehensive machine learning analyses were performed on 70 candidate targets, generating 127 prediction models to identify core genes associated with CBD-related hypertension. The Lasso + RF integrated model showed optimal performance on both the training and validation sets (Fig. 4A), and 10 feature genes were identified. A protein–protein interaction (PPI) network of these feature genes, constructed using the GeneMANIA platform, revealed their close association with biological processes including sprouting angiogenesis, cellular response to peptides, calcium-release channel activity, and cardiac conduction (Fig. 4C). ROC curve analysis across training and testing groups indicated that PKM possessed high sensitivity and specificity for distinguishing hypertensive patients from healthy individuals (AUC > 0.95). Four additional genes (THRB, AKR1B1, TGFBR1, SRC) met the diagnostic potential criterion (AUC > 0.85). Together with PKM, these five genes were identified as core targets for CBD intervention in HTN (Fig. 4B), and their differential expression in hypertensive patient blood was visualized in a volcano plot (Fig. 4D). SHAP interpretability analysis revealed distinct functional contributions of the core genes. PKM exerted the largest influence (SHAP value = 0.293) and was the strongest predictor (Fig. 4E). TGFBR1 and the other four core genes exhibited bidirectional regulatory effects (Fig. 4F). A SHAP density plot illustrated the distribution of gene SHAP values across groups, showing that the core genes contributed negatively to predictions for the Control group and positively to predictions for the Treatment group (Fig. 4G). Force-directed analysis showed that PKM (25.8, Δ = + 0.261), as the primary positive regulator, raised the predicted value (f(x) = 1) above the baseline expected value (E[f(x)] = 0.578) (Fig. 4H). Nonlinear relationships among the core genes were also identified: PKM reached its peak predictive contribution at high expression levels (Expression Levels > 27), and TGFBR1 expression correlated negatively with SRC expression (Fig. 4I).

### Analysis of the shortest interaction paths of core genes

**Fig. 5:**
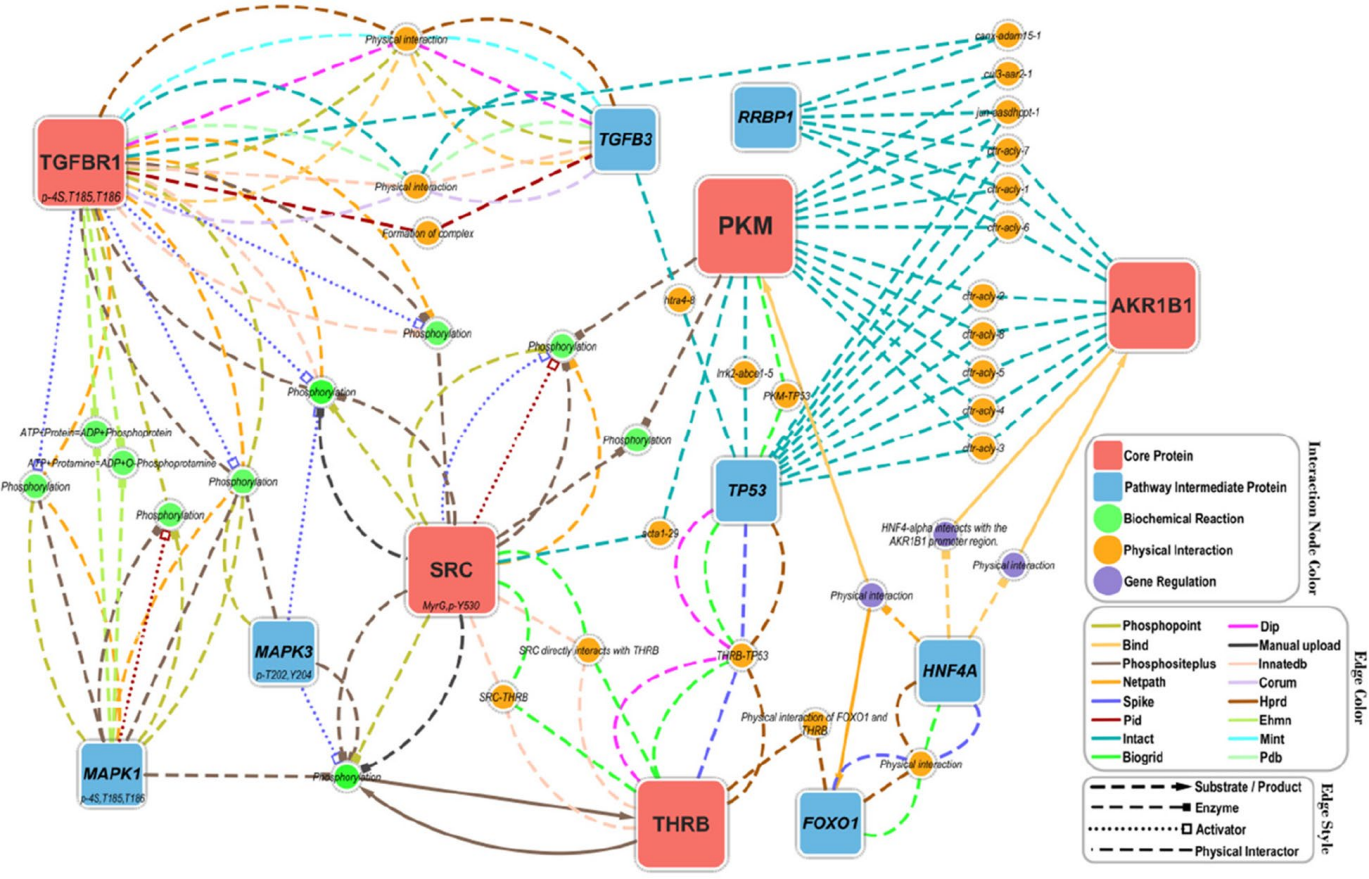
Analysis of the shortest interaction paths and regulatory relationships among core genes. Red rectangles denote core proteins, while blue rectangles denote pathway intermediate proteins that participate in specific signaling or metabolic pathways. Green circles indicate metabolic or modification reactions (for example, phosphorylation), and orange circles indicate direct protein–protein binding (for example, receptor–ligand or enzyme–substrate interactions). Purple circles indicate regulation of gene expression by transcription factors (for example, activation or inhibition). Edge style specifies the functional type of each interaction between nodes (Substrate/Product, Enzyme, Activator, or Physical Interactor). Edge colors indicate interaction data sources, including Phosphopoint (phosphorylation site database), Bind (binding experiments), Netpath (signaling pathway database), Biogrid/Intact (protein interaction databases), Manual upload, and others

The shortest interaction paths among the core genes (PKM, THRB, AKR1B1, TGFBR1, SRC) were analyzed using the ConsensusPathDB database. Pathway proteins and biochemical reactions were linked by physical binding, phosphorylation, metabolic reactions, and other mechanisms that regulate cell signaling, metabolism, and gene expression. Edge style and color indicate interaction types and data sources, which aid in the assessment of interaction reliability. The results showed direct interactions between PKM and AKR1B1, PKM and SRC, THRB and SRC, and TGFBR1 and SRC; the remaining proteins were regulated indirectly through pathway intermediates such as Cellular tumor antigen p53 (TP53), Mitogen-activated protein kinase 1 (MAPK1), Mitogen-activated protein kinase 3 (MAPK3), Ribosome-binding protein 1 (RRBP1), Forkhead box protein O1 (FOXO1), Hepatocyte nuclear factor 4-alpha (HNF4A), and Transforming growth factor beta-3 proprotein (TGFB3) (Fig. 5).

### Analysis of immune cell infiltration

**Fig. 6:**
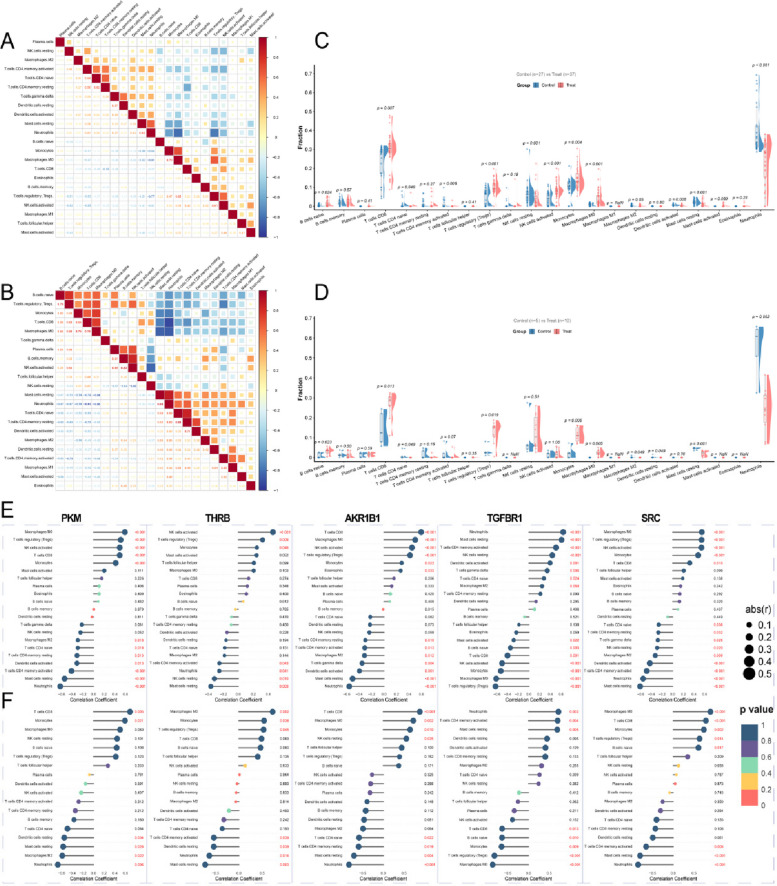
Immune characteristics associated with HTN and immune-infiltrating cells related to core genes. **A**, **B** Correlation analysis of key immune cell types in the training and validation sets. **C**, **D** Raincloud plots comparing levels of 22 immune-infiltrating cell types between the training and validation sets. **E**, **F** Lollipop plots showing correlations between core genes and immune-infiltrating cells in the training and validation sets

Neutrophils and resting mast cells showed a marked decrease in infiltration in both the HTN training and validation cohorts. Their infiltration levels correlated negatively with the core genes PKM, THRB, AKR1B1, and SRC and positively with TGFBR1 (Fig. 6). These findings indicate that neutrophils and resting mast cells play key roles in the immune-related pathways regulated by these core genes.

### Molecular docking validation of CBD–core gene interactions

Potential binding between CBD and the core genes (PKM, THRB, AKR1B1, TGFBR1, and SRC) was validated through comprehensive molecular docking analyses. The docking produced binding energies of −9.2, −9.4, −8.0, −10.3, and −8.6 kcal/mol for CBD with PKM, THRB, AKR1B1, TGFBR1, and SRC, respectively, indicating strong molecular interactions between CBD and these proteins (Hsin et al. 2013).

**Fig. 7:**
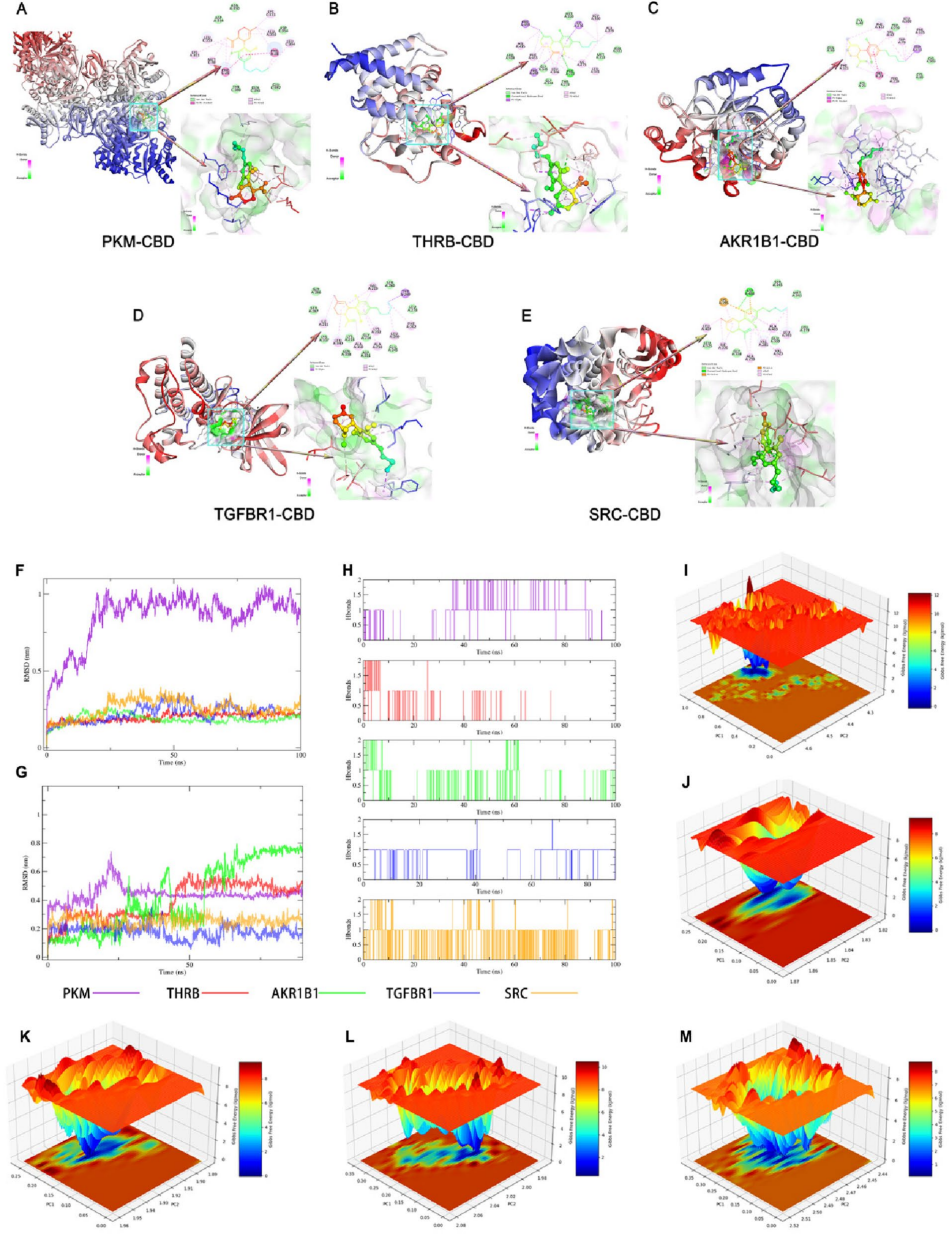
Molecular docking validation of CBD–core gene interactions. **A** Docking results for PKM with CBD. **B** Docking results for THRB with CBD. **C** Docking results for AKR1B1 with CBD. **D** Docking results for TGFBR1 with CBD. **E** Docking results for SRC with CBD. **F**, **G** RMSD profiles of the CBD–protein complexes during the 100-ns molecular dynamics simulations, showing target-specific conformational stability and structural adaptation. **H** Hydrogen-bond occupancy analysis of the five CBD–protein complexes over the simulation trajectory. **I-M** Free energy landscapes of the CBD–PKM, CBD–THRB, CBD–AKR1B1, CBD–TGFBR1, and CBD–SRC complexes, respectively, were generated from principal component analysis using PC1 and PC2

Binding pocket analysis indicated that CBD interacted with chemically compatible cavities through target-specific combinations of hydrophobic, π-related, and polar contacts. Briefly, PKM and AKR1B1 displayed interaction patterns dominated by aromatic/hydrophobic stabilization, centered on representative residues such as Phe26 and Trp20, respectively. THRB and TGFBR1 accommodated CBD within predominantly hydrophobic pockets lined by residues, including Phe/Ile/Leu-rich cavity components, consistent with their shapes and hydrophobic complementarity. In contrast, SRC showed a mixed pocket environment in which hydrophobic packing was accompanied by additional polar/electrostatic interactions involving residues such as Lys295 and Asp404 (Fig. 7A-E).

To assess the stability of the five CBD-bound complexes, a 100 ns molecular dynamics simulation was conducted. RMSD analyses revealed target-dependent dynamic behaviors: CBD–PKM exhibited the largest structural fluctuations, then stabilized after a notable conformational rearrangement, whereas CBD–THRB, CBD–TGFBR1, and CBD–SRC maintained consistently low deviations, indicating greater stability. CBD–AKR1B1 showed intermediate behavior with gradual conformational adaptation (Fig. 7F-G). Hydrogen-bond analysis further highlighted distinct interaction patterns: CBD–SRC showed the highest persistence, whereas CBD–AKR1B1 and CBD–TGFBR1 formed intermittent yet recurrent hydrogen bonds. In contrast, CBD–PKM and CBD–THRB exhibited fewer stable hydrogen bonds, suggesting a greater reliance on hydrophobic and π-mediated interactions (Fig. 7H). Principal component analysis-derived free energy landscapes indicated convergence toward energetically favorable states across all systems, albeit with varying conformational sampling. CBD–PKM and CBD–THRB populated relatively confined low-energy basins, whereas CBD–AKR1B1, CBD–TGFBR1, and CBD–SRC explored broader low-energy regions, reflecting multiple metastable conformations (Fig. 7I-M). Collectively, these findings support the structural plausibility and dynamic stability of CBD binding while revealing target-specific conformational landscapes.

## Discussion

In this study, we integrated multi-omics datasets with machine learning, bioinformatics, and immune-regulation analyses to identify molecular targets through which CBD exerts its effects in HTN. Bioinformatics analysis revealed 70 candidate genes, and a machine learning model selected 10 feature genes. A PPI regulatory network indicated that these genes are closely associated with sprouting angiogenesis and calcium-release channel activity. Five core genes (PKM, THRB, AKR1B1, TGFBR1, SRC) stood out, and the model confirmed their strong diagnostic value (ROC > 0.85). Differential expression analysis showed that PKM, THRB, AKR1B1, and SRC protein levels were elevated in the blood of HTN patients, whereas TGFBR1 expression was decreased. SHAP interpretability analysis indicated that PKM (SHAP value = 5.8, Δ = + 0.261) was the strongest positive regulatory predictor. ConsensusPathDB analysis confirmed direct interactions among the core genes. CIBERSORT and regulatory network analysis revealed mutual regulatory relationships between neutrophil and resting mast cell infiltration and the core genes. Molecular docking simulations supported the biological relevance of these findings by showing strong binding affinity between CBD and core gene products mediated by specific amino acid interactions. Collectively, these results suggested that the identified core genes may serve as key molecular targets for CBD intervention in HTN.

The identified core genes play multiple roles in the mechanism by which CBD exerts its effects on hypertension. Pyruvate kinase M2 (PKM2), encoded by the PKM gene, is a candidate metabolic switch that drives hypertensive vascular remodeling (Wu et al. 2022). PKM2 promotes glycolytic reprogramming of vascular smooth muscle cells and their conversion to a synthetic phenotype (Cao et al. 2025). Recent studies have provided critical evidence: in an angiotensin II–induced hypertension model, the aorta showed a marked increase in glycolytic flux, accompanied by vascular hypertrophy and fibrosis (Dikalova et al. 2025). Manish Jain et al. further supported this mechanism: specific PKM2 knockout in vascular smooth muscle cells effectively inhibited their shift from a contractile to a synthetic phenotype (Jain et al. 2021). c-Src, encoded by the SRC gene, is activated by mechanical stress or angiotensin II. It then enhances VSMCs' contraction, proliferation, and migration via signaling pathways such as MAPK1/3 and p38 MAPK, thereby promoting small artery remodeling and increasing peripheral resistance (Rice et al. 2002; Touyz et al. 2001). Clinical evidence shows that NOX5-derived reactive oxygen species in VSMCs from hypertensive patients form a positive feedback loop through c-Src, thereby exacerbating contractile signaling; this process is partially reversible with c-Src inhibitors (Camargo et al. 2022; Callera et al. 2016). In this context, the observed upregulation of PKM\SRC in HTN blood samples is directionally consistent with a pathogenic role in hypertension-associated metabolic remodeling. By contrast, TGFBR1, THRB, and AKR1B1 warrant cautious interpretation. TGFBR1, the principal type I receptor of the TGF-β pathway, regulates fibrosis and extracellular matrix remodeling in hypertension. Activation of TGF-β promotes the production of collagen and fibronectin by vascular smooth muscle cells (VSMCs). It drives fibroblast differentiation into myofibroblasts, collectively contributing to vascular wall thickening, lumen narrowing, and reduced compliance (August and Suthanthiran 2006). THRB mediates thyroid hormone (T3) signaling, influencing metabolic and cardiovascular homeostasis (Brent 2023; Cui et al. 2021). AKR1B1 encodes aldose reductase, and the C-106T polymorphism modulates enzyme activity, with variable allele frequency across hypertensive populations (Wang et al. 2016).

Analysis of the shortest interaction paths among core genes revealed several pathway intermediate proteins: TP53, MAPK1, MAPK3, RRBP1, FOXO1, HNF4A, and TGFB3. These proteins also play crucial roles in the progression of hypertension. Loss of function of the MAPK1 and MAPK3 signaling pathways can cause endothelial dysfunction, which activates TGFβ signaling, induces endothelial-to-mesenchymal transition and fibrosis, and leads to hypertension and end-organ damage (Ricard et al. 2019). As a key effector in this cascade, TGFB3 expression directly affects vascular smooth muscle contraction and vascular tension (Badri et al. 2013). For vascular protection, TP53 forms a transcriptional complex with PPARγ to activate protective gene programs under stress, and impaired TP53 function is closely associated with vascular lesions (Hennigs et al. 2021).RRBP1 directly influences renin secretion and renin–angiotensin–aldosterone system activity by promoting prorenin transport between the endoplasmic reticulum and the Golgi apparatus, thereby determining blood pressure levels (Chiu et al. 2023). Dehydroepiandrosterone activates FOXO1 phosphorylation via the mineralocorticoid receptor pathway, thereby upregulating endothelin-1 expression, which promotes vasoconstriction and raises blood pressure (Lindschau et al. 2011). Moreover, HNF4A modulates the local renin–angiotensin system by directly regulating Angiotensin-Converting Enzyme 2 expression (Xu et al. 2024).

Associations between CBD–HTN characteristic genes and immune cell infiltration were examined using the CIBERSORT algorithm. Neutrophil and mast cell infiltration are known to play important roles in the vascular pathology of hypertension. Neutrophils may contribute to hypertension progression, in part by forming neutrophil extracellular traps (NETs), which have been associated with vascular inflammation and endothelial dysfunction (Krishnan et al. 2024). In parallel, mast cells in the hypertensive microenvironment may be activated and degranulate, releasing mediators such as histamine, proteases, and cytokines that contribute to vascular remodeling and blood pressure elevation (Ge et al. 2022). From this perspective, A lower neutrophil fraction may indicate a shift in inflammatory status during hypertension progression. At the same time, a decrease in resting mast cells may be associated with changes in the mast-cell activation state (Krishnan et al. 2022; Poto et al. 2024). Our results suggest that PKM, THRB, AKR1B1, and SRC were inversely associated with the estimated fractions of neutrophils and resting mast cells, whereas TGFBR1 showed a positive association. These relationships may indicate a link between the identified characteristic genes and hypertension-related immune remodeling. Such findings are more appropriately understood as transcriptome-based inferences, since CIBERSORT estimates relative immune-cell composition from bulk expression profiles rather than directly measuring cell abundance or functional state, especially in peripheral whole-blood samples.

We acknowledge several key limitations of the present study that are critical to note when interpreting our findings. First, currently publicly available hypertension transcriptomic datasets are constrained by both limited sample size and generally poor completeness of clinical annotation. Even after including all eligible public cohorts, we were still unable to perform fully adequate covariate adjustment and fine-grained subgroup analyses. Furthermore, as the same cohort was used for both model development and subsequent phenotype stratification assessment, the validation framework of this study is not fully independent, which may have introduced a degree of optimism bias into the performance estimation of our predictive model. Meanwhile, the core analyses of this study are primarily based on transcriptomic data derived from peripheral blood. Since the pathogenesis of hypertension involves multiple target tissues, including the vascular wall, kidney, heart, and immune microenvironment, transcriptomic features from peripheral blood cannot fully recapitulate disease-specific biological alterations in these hypertension-affected tissues. Finally, all CBD-associated candidate targets included in this study were predicted via in silico machine learning simulations; molecular dynamics simulations only provide supportive structural validation evidence, which is insufficient to directly confirm the binding specificity between CBD and its candidate targets, nor can this approach definitively establish the causal pharmacological effects of this interaction. Accordingly, the findings reported herein should be considered preliminary and hypothesis-generating, and require further validation in independent sample cohorts and experimental systems. Despite these limitations, by integrating multimodal analytical strategies combining transcriptomic analysis, network-based screening, machine learning, immune infiltration assessment, and molecular dynamics simulations, this study provides a valuable reference framework for further investigation into the underlying molecular mechanisms linking cannabidiol and hypertension.

## Conclusion

In conclusion, this study identified several candidate genes potentially associated with the CBD–hypertension molecular network, including PKM, THRB, AKR1B1, TGFBR1, and SRC. Integrated bioinformatics analyses and structural modeling suggested that these genes may participate in hypertension-related biological processes. However, because the present findings are based primarily on computational analyses, they should not be interpreted as confirmation of direct therapeutic targets or as evidence for immediate clinical application. Additional experimental and translational studies are needed to validate these associations, clarify the underlying mechanisms, and determine the relevance of these targets to the potential antihypertensive effects of CBD.

## Data Availability

The data used are from the comprehensive database (Gene Expression Omnibus), with accession numbers: GSE234085, GSE236442, GSE308365, and GSE262814. They can be accessed through the following website: https://www.ncbi.nlm.nih.gov/geo/.

## Abbreviations

AKR1B1: Aldo-keto reductase family 1 member B1
AUC: Area under the curve
CBD: Cannabidiol
CIBERSORT: Cell-type Identification By Estimated Regression of SORTing
DAVID: Database for Annotation, Visualization and Integrated Discovery
DEGs: Differentially expressed genes
Enet: Elastic Net
FOXO1: Forkhead box protein O1
GBM: Gradient Boosting Machine
glmBoost: Generalized Linear Model with Boosting
GO: Gene Ontology
HNF4A: Hepatocyte nuclear factor 4-alpha
HTN: Hypertension
KEGG: Kyoto Encyclopedia of Genes and Genomes
Lasso: Least Absolute Shrinkage and Selection Operator
LDA: Linear Discriminant Analysis
MAPK1: Mitogen-activated protein kinase 1
MAPK3: Mitogen-activated protein kinase 3
NETs: Neutrophil extracellular traps
PKM: Pyruvate kinase PKM
plsRglm: Partial Least Squares Regression for GLM
PPI: Protein-protein interaction
RF: Random Forest
Ridge: Ridge Regression
RRBP1: Ribosome-binding protein 1
SHAP: SHapley Additive exPlanations
SRC: Proto-oncogene tyrosine-protein kinase Src
Stepglm: Stepwise Generalized Linear Model
SVA: Surrogate variable analysis
SVM: Support Vector Machine
TGFB3: Transforming growth factor beta-3 proprotein
TGFBR1: TGF-beta receptor type-1
THRB: Thyroid hormone receptor beta
TOM: Topological overlap matrix
TP53: Cellular tumor antigen p53
VSMCs: Vascular smooth muscle cells
WGCNA: Weighted gene co-expression network analysis
XGBoost: EXtreme Gradient Boosting
